# Prognostic value of metabolic syndrome in patients with heart failure and malnutrition

**DOI:** 10.1186/s12872-024-03767-5

**Published:** 2024-03-02

**Authors:** Xuehe Zhang, Chunfang Shan, Kaixuan Hu, Binbin Fang, Zhiyang Zhang, Qian Xie, Chang Liu, Xin An, Yining Yang, Xiaomei Li

**Affiliations:** 1 Department of Cardiology, First Affiliated Hospital of Xinjiang Medical University, 137 Liyushan South Road, Urumqi, 830054 People’s Republic of China; 2 Department of Cardiology, Bayinguoleng Mongolian Autonomous Prefecture People’s Hospital, Korla, China; 3 State Key Laboratory of Pathogenesis, Prevention and Treatment of High Incidence Diseases in Central Asia, Clinical Medical Research Institute, First Affiliated Hospital of Xinjiang Medical University, Urumqi, China; 4 Department of Cardiology, People’s Hospital of Xinjiang Uygur Autonomous Region, 91 Tianchi Road, Urumqi, 830054 People’s Republic of China

**Keywords:** Prognostic nutritional index, Cardiovascular death, Malnutrition

## Abstract

**Background:**

Malnutrition is severely associated with worst prognosis of patients with heart failure (HF). Malnourished patients with the metabolic syndrome (MS) can result in a double burden of malnutrition. We aimed to investigate the impact of the MS on clinical outcomes in malnourished HF patients.

**Methods:**

We examined 529 HF patients at risk of malnutrition with a mean age of (66 ± 10) years and 78% (415) were male. Nutritional status defined primarily by the prognostic nutritional index (PNI), with PNI < 40 being defined as malnutrition. The follow-up endpoint was cardiovascular death or all-cause death.

**Results:**

During the 36-month follow-up, survival rates for cardiovascular and all-cause death were significantly lower in the MS group than in the non-MS group (log-rank *P* < 0.01). Multivariate Cox proportional hazards regression models showed that MS was independently associated with cardiovascular death (HR:1.759, 95%CI:1.351–2.291, *p* < 0.001) and all-cause death (HR:1.326, 95%CI:1.041–1.689, *p* = 0.022) in malnourished patients with HF. MS significantly increased the predictive value of cardiovascular death (AUC:0.669, 95%CI:0.623–0.715, *p* < 0.001) and all-cause death (AUC:0.636, 95%CI:0.585–0.687, *p* < 0.001) on the basis of established risk factors. The predictive effect of MS on cardiovascular death was independent of sex, age, functional class and left ventricular ejection fraction.

**Conclusions:**

In malnourished patients with HF, MS is an independent risk factor for cardiovascular and all-cause mortality. MS significantly enhance the predictive value for clinical events in patients.

**Supplementary Information:**

The online version contains supplementary material available at 10.1186/s12872-024-03767-5.

## Introduction

Heart failure (HF) is a manifestation of various end-state of heart diseases [1, 2].The prevalence of HF, a serious public health problem, is increasing every year. The mortality rate of HF is high and seriously affects the quality of life of patients [3]. As with many chronic diseases, up to 50% of patients with HF have some form of malnutrition in combination [4]. Patients with HF suffer from inadequate intake due to liver congestion, intestinal edema, and impaired absorption, but at the same time cardiac metabolism and energy demands increase. This results in an imbalance between energy intake and expenditure [5]. Malnutrition increases the length of hospital stay as well as the risk of rehospitalization and death in patients with cardiovascular disease and is considered to be an important influencing factor in poor patient prognosis [6–8]. Appropriate nutritional interventions can reduce cardiovascular disease risk and improve clinical outcomes in patients with HF [9]. In recent years, nutritional status has attracted attention as a modifiable risk factor for patients with HF [10–13]. Routine nutritional screening is recommended for most patients with chronic and acute illnesses and for hospitalized patients to identify those at risk of malnutrition. Current guidelines for the management of HF also recommend the assessment of nutritional status in patients with chronic HF [14, 15]. However, to a large extent, nutrition awareness and intervention by clinicians are inadequate. To date, there is no standardized method to determine the nutritional risk of HF patients. The prognostic nutritional index (PNI) is calculated from serum albumin and lymphocyte counts and can be used to detect cardiometabolic disorders in patients with HF, allowing for early detection of malabsorption and inflammatory disease. At present, PNI has been widely used as a method to assess nutritional status. Previous studies have shown that PNI is independently associated with adverse clinical outcomes in patients with acute HF with different levels of ejection fraction [16]. Subsequent studies have found that PNI can independently predict the prognosis of patients with severe decompensated acute HF [17].

Malnutrition is widespread and takes many forms. Overproduction and underproduction are usually the two directions of malnutrition. The double burden of malnutrition is manifested by the interaction of undernutrition and overweight/obesity, two forms of malnutrition that share many common drivers and have adverse effects on human health [18]. . Going further, the double burden concept of malnutrition has also been proposed to extend to the individual. Obesity is the most common component of the metabolic syndrome (MS), which implies that overnutrition contributes to the syndrome. Obesity is an established risk factor for cardiovascular disease. Malnutrition may exacerbate the cardiac effects associated with chronic morbid obesity, and studies have shown that malnourished obese individuals have maladaptive cardiac remodeling and the worst cardiac outcomes [19]. Most patients with MS present with obesity, which implies that overnutrition contributes to the syndrome [20]. MS refers to a range of metabolism-related disorders, including glucose intolerance, insulin resistance, obesity, dyslipidemia, and type 2 diabetes [21–23]. As a health problem in modern society, MS is associated with a huge social, personal, and economic burden in both developing and developed countries. Currently, it is calculated that MS affects approximately 25% of the global population [24, 25].

To our knowledge, there are no relevant studies focusing on the impact of MS in malnourished HF patients. Many relevant studies have highlighted the importance of nutritional assessment in clinical practice, especially for the health management of end-stage patients with cardiac dysfunction [9, 26]. Importantly, malnutrition not only refers to wasting/under-nutrition but also overweight/obesity [18]. The prognosis of obese critically ill patients with malnutrition is worse compared to those without malnutrition [27]. The aim of this study was to assess the impact of MS on the prognosis of malnourished HF patients.

## Methods

### Study design and subjects

This study was a single-center retrospective study. HF patients at risk of malnutrition who met the diagnostic criteria of MS and sex- and age-matched nonMS patients were enrolled in the Heart Center of the First Affiliated Hospital of Xinjiang Medical University from January 2015 to December 2019. Most of the patients were admitted to the hospital because of common clinical manifestations of heart failure such as chest tightness and dyspnea, aggravation of pre-existing heart failure symptoms, or following the doctor’s instructions for regular review. MS was defined as any three or more of the following: waist circumference > 102 cm in men, > 88 cm in women; blood pressure > 130/85 mmHg or on medication; fasting plasma glucose (FPG) ≥ 110 mg/dL or on medication; triglyceride (TG) level ≥ 150 mg/dL; high-density lipoprotein cholesterol (HDL-C) < 40 mg/dL for men and < 50 mg/dL for women [28]. The exclusion criteria were as follows: patients with autoimmune diseases, acute infectious diseases, severe liver and kidney dysfunction, malignant tumors, hematological diseases, incomplete clinical data, and inability to complete the follow-up. The clinical information of the subjects was collected by specialized personnel blinded to the purpose of the study and included data regarding sex, age, smoking, drinking, past history, and diagnosis. Blood samples were collected at admission and tested by the Laboratory Center of Xinjiang Medical University, and blood pressure was measured at admission. Echocardiography was performed within 1 week after admission to measure left ventricular ejection fraction (LVEF). All study participants provided written informed consent. The study was approved by the Ethics Review Committee of the First Affiliated Hospital of Xinjiang Medical University (20141201-03-1701 A). The study conformed to the principles outlined in the Declaration of Helsinki.

### Definitions of clinical characteristics

In this study, PNI was used to assess nutritional status, PNI = serum albumin (ALB) (g/L) + 5×lymphocyte count(LYM) (10^9^/L) [29]. PNI < 40 was defined as the presence of malnutrition risk [30, 31]. Body mass index (BMI) was calculated as weight in kilograms divided by the square of height in meters (kg/m2). The Cockcroft-Gault equation was used to calculate estimated glomerular filtration rate (eGFR), eGFR=[(140-age) ×weight(kg)] ×0.85(if female) / [72×serum creatinine(mg/dl)] [32].

### Clinical outcome and follow-up

We investigated clinical outcomes over a 36-month follow-up period after discharge by means of an outpatient questionnaire or telephone interview. The endpoint of follow-up was defined as cardiovascular death or all-cause death. Cardiovascular death was defined as death due to myocardial infarction, HF, arrhythmia, or cardiac-related surgery.

### Statistical analysis

Statistical analyses were performed using SPSS Statistics version 25.0 (IBM Corporation, Chicago, IL, USA) and the R statistical programming language version 4.1.2 (Vienna, Austria). Continuous variables are expressed as the means with standard deviations or as medians with interquartile ranges. The t test or Mann‒Whitney U test was used for comparisons between groups. Categorical variables were expressed as frequencies and compared between groups using the chi-square test. Unadjusted survival rates were estimated using Kaplan‒Meier curves and compared between groups using the log-rank test. Multivariate Cox proportional hazards regression analysis was used to determine the effect of MS on clinical outcomes in malnourished patients with HF. Receiver operating characteristic (ROC) curve analysis was used to evaluate the effect of MS addition on the prediction of adverse events, and the area under the curve (AUC) was compared. Subgroup analysis was used to analyze the relationship between MS and cardiovascular death in malnourished patients with HF according to age, sex, New York Heart Association (NYHA) class and LVEF. A *p* value (two-tailed)<0.05 was considered significant.

## Results

### Study population and baseline characteristics

A total of 594 patients were included in this study with a loss to follow-up rate of 10.9%. Finally 529 patients completed 36 months of follow-up with a mean age of (66 ± 10) years and 78% (415) were male. PNI of all patients was less than 40. Patients were grouped according to whether they had combined MS or not: the MS group and the non-MS group. The clinical baseline characteristics of the different groups of patients are shown in Table 1. There were more patients with previous stroke in the non-MS group. BMI, systolic blood pressure (SBP), and diastolic blood pressure (DBP) were significantly higher in the MS group than in the non-MS group, and NYHA class IV patients were higher in the MS group than in the non-MS group. In terms of laboratory findings, the levels of white blood cell count (WBC), neutrophil count (NEUT), MONO, FPG, TG and B-type natriuretic peptide (BNP) in the MS group were significantly higher than those in the non-MS group, and the eGFR level was significantly lower than that in the non-MS group. HDL-C in the non-MS group was significantly higher than that in the MS group. There were no significant differences in other variables between the two groups.

**Table 1:** Baseline characteristics of patients in different groups

Variable	MS(*n* = 221)	Non-MS(*n* = 308)	*p*
PNI	36.25(33.50,38.25)	36.45(34.88,38.31)	0.201
Age, years	66 ± 10	66 ± 10	0.909
Male, n(%)	169(76.5)	246(79.9)	0.880
Smoking, n(%)	90(40.7)	138(44.8)	0.350
Drinking, n(%)	55(24.9)	79(25.6)	0.842
Previous MI, n(%)	60(27.1)	96(31.2)	0.317
Previous stroke, n(%)	24(10.9)	150(48.7)	< 0.001
BMI, kg/m^2^	26.17 ± 3.78	23.37 ± 3.09	< 0.001
WC, cm	97.6 ± 11.0	88.8 ± 9.7	< 0.001
NYHA class			< 0.001
I	0(0)	0(0)	
II	21(9.5)	52(16.9)	
III	105(47.5)	187(60.7)	
IV	95(43.0)	69(22.4)	
SBP, mmHg	129 ± 22	122 ± 27	0.002
DBP, mmHg	76 ± 13	73 ± 12	0.002
WBC,10^9^/L	7.10(5.75,9.56)	6.71(5.63,8.07)	0.013
NEUT,10^9^/L	5.08(3.87,7.27)	4.35(3.41,5.63)	< 0.001
LYM,10^9^/L	1.10(0.81,1.40)	1.14(0.82,1.57)	0.201
MONO,10^9^/L	0.58(0.44,0.73)	0.53(0.40,0.68)	0.032
FPG, mg/dL	154.98(110.70,241.02)	142.38(100.98,210.69)	0.032
TG, mg/dL	106.32(80.63,147.08)	95.25(69.11,122.71)	< 0.001
TC, mg/dL	121.13(102.94,155.19)	131.19(108.36,154.41)	0.058
HDL-C, mg/dL	29.41(23.99,37.15)	40.64(31.35,48.76)	< 0.001
LDL-C, mg/dL	78.95(61.15,102.94)	82.24(63.86,106.81)	0.484
eGFR, mL/min/1.73 m^2^	54.40(33.78,81.42)	66.63(49.14,85.31)	< 0.001
ALB, g/L	30.50(27.40,32.60)	30.48(28.50,32.40)	0.562
LVEF, %	43(37,49)	40(37,46)	0.061
BNP, pg/ml	4498.00(1727.00,10023.00)	2852.50(1054.73,5870.00)	< 0.001

PNI, prognostic nutritional index; MI, myocardial Infarction; BMI, body mass index; WC, waist circumference; NYHA, New York Heart Association; SBP, systolic blood pressure; DBP, diastolic blood pressure; WBC, white blood cell count; NEUT, neutrophil count; LYM, lymphocyte count; MONO, monocyte count; FPG, fasting plasma glucose; TG, triglyceride; TC, total cholesterol; HDL-C, high-density lipoprotein cholesterol; LDL-C, low-density lipoprotein cholesterol; eGFR, estimated glomerular filtration rate; ALB, serum albumin; LVEF, left ventricular ejection fraction; BNP, brain natriuretic peptide

### Effect of MS on adverse outcomes

**Fig. 1:**
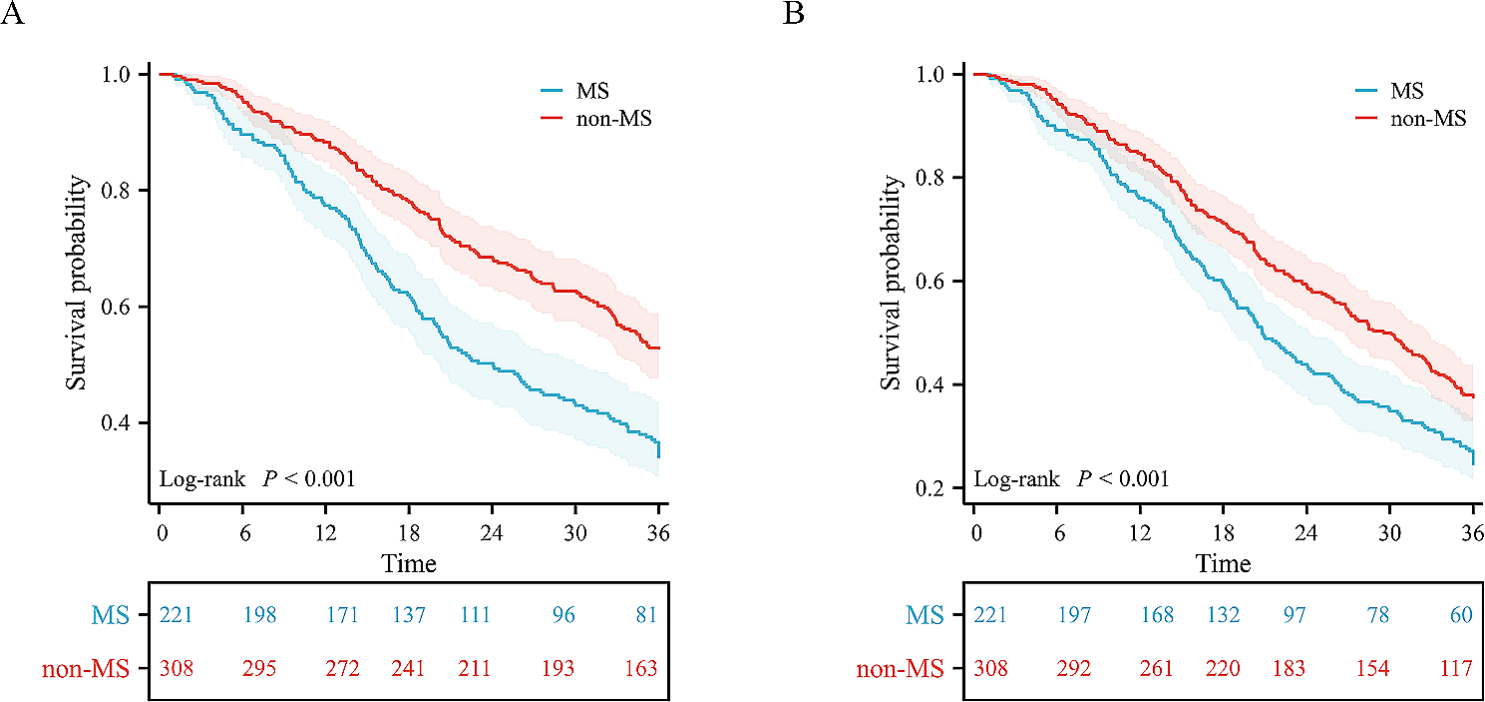
Kaplan‒Meier analysis of adverse outcomes in different groups. (**A**) Cardiovascular death. (**B**) All-cause death

During the follow-up period, cardiovascular death occurred in 292 patients (55%), and all-cause death occurred in 360 patients (68%). Kaplan-Meier analysis showed that the presence of MS significantly reduced survival probability in malnourished patients with HF (log-rank *p* < 0.05) (Fig. 1). We used Cox proportional hazard regression models to determine the association between MS and adverse outcomes in malnourished and HF patients. In the unadjusted Cox model, MS was a predictor of cardiovascular death (HR = 1.723, 95% CI: 1.369–2.168, *p* < 0.001) and all-cause death (HR = 1.464, 95% CI: 1.189–1.801, *p* < 0.001) in malnourished HF patients. Model 1 was adjusted for sex and age, and Model 2 was further adjusted for smoking, drinking, previous MI, previous stroke and NYHA class based on Model 1. Model 3 was further adjusted for low-density lipoprotein cholesterol (LDL-C), eGFR, LVEF, and BNP on the basis of Model 2. The results are shown in Table 2. After adjusting for other covariables, we found that MS was also independently associated with cardiovascular death (HR: 1.759, 95%: 1.351–2.291, *p* < 0.001) and all-cause death (HR: 1.326, 95%: 1.041–1.689, *p* = 0.022) in patients with malnourished HF.

**Table 2:** Unadjusted and adjusted Cox regression analysis of adverse outcomes

	Non-MS	MS HR (95%CI)	*p*
Cardiovascular death			
Unadjusted	Reference	1.723(1.369–2.168)	< 0.001
Model1	Reference	1.723(1.369–2.168)	< 0.001
Model2	Reference	1.734(1.331–2.260)	< 0.001
Model3	Reference	1.759(1.351–2.291)	< 0.001
All cause death			
Unadjusted	Reference	1.464(1.189–1.801)	< 0.001
Model1	Reference	1.464(1.190–1.802)	< 0.001
Model2	Reference	1.324(1.042–1.683)	0.022
Model3	Reference	1.326(1.041–1.689)	0.022

Model1: Age, Sex. Model2: Age, Sex, Smoking, Drinking, Previous MI, Previous Stroke, NYHA class. Model3: Age, Sex, Smoking, Drinking, Previous MI, Previous Stroke, NYHA class, LDL-C, eGFR, LVEF, BNP. HR: hazard ratio; CI: confidence interval

### Predictive value of MS

Established risk factors including age, sex, smoking, drinking, eGFR, LVEF, BNP, NYHA class, previous Stroke, previous MI. Established risk factor model had significant predictive value for long-term cardiovascular death (AUC: 0.632, 95% CI: 5585 − 0.679, *p* < 0.001) and all-cause death in patients with malnourished HF (AUC: 0.626, 95%: 0.576–0.677, *p* < 0.001). Adding an MS diagnosis to the established risk factor model significantly improved the prediction of cardiovascular death in malnourished HF patients, increasing the AUC from 0.632 to 0.669. The addition of MS also increased the predictive value for all-cause death (Table 3). Subgroup analysis for age, sex, NYHA class, and LVEF showed that after adjusting for age, sex, smoking, drinking, previous myocardial infarction (MI), previous stroke, NYHA class, eGFR, BNP, LVEF, and LDL-C, MS remained an independent predictor of cardiovascular death in patients with malnourished HF (Table 4).

**Table 3:** Prognostic value of metabolic syndrome

	AUC (95%CI)	*p*
Cardiovascular death		
Established risk factors	0.632(0.585–0.679)	< 0.001
+MS	0.669(0.623–0.715)	< 0.001
All cause death		
Established risk factors	0.626(0.576–0.677)	< 0.001
+MS	0.636(0.585–0.687)	< 0.001

Established risk factors: Age, Sex, Smoking, Drinking, eGFR, LVEF, BNP, NYHA class, Previous Stroke, Previous MI. AUC, area under the curve

**Table 4:** Prognostic value of metabolic syndrome for cardiovascular death in different subgroups

Subgroups	Case	Unadjusted HR(95%CI)		*p*	Adjusted HR(95%CI)		*p*
Age							
≥ 60	389	1.735(1.328–2.267)		< 0.001	1.660(1.224–2.251)		0.001
< 60	140	1.630(1.065–2.493)		0.024	1.969(1.184–3.274)		0.009
Gender							
Male	415	1.568(1.208–2.035)		0.001	1.556(1.158–2.090)		0.003
Female	114	2.423(1.476–3.977)		< 0.001	4.075(2.012–8.251)		< 0.001
NYHA class							
II-III	365	1.731(1.305–2.295)		< 0.001	1.805(1.324–2.460)		< 0.001
IV	164	1.760(1.138–2.722)		0.011	1.780(1.018–3.111)		0.043
LVEF							
≥ 40	305	1.625(1.203–2.195)		0.002	1.877(1.399–2.629)		< 0.001
< 40	224	1.780(1.257–2.521)		0.001	1.668(1.062–2.619)		0.026

Adjusted variables were age, sex, smoking, drinking, previous MI, previous Stroke, NYHA class, eGFR, BNP, LVEF, LDL-C. HR: hazard ratio; CI: confidence interval

## Discussion

In our study, PNI was selected to assess the nutritional status of patients with HF, and low PNI levels were taken as evidence of malnutrition. Follow-up of malnourished HF patients showed that patients with MS had a worse prognosis. The survival probability of patients with MS is significantly lower than that of patients without MS. In malnourished HF patients, MS was independently associated with cardiovascular death and all-cause death.

Malnutrition is very common in patients with HF. Malnutrition occurs in patients with HF through a number of mechanisms due to intestinal edema and impaired intestinal barrier function, increased energy expenditure and decreased anabolism, and chronic inflammatory states [5, 33, 34]. Patients with advanced chronic HF often develop cardiac cachexia, which manifests as muscle wasting and weight loss. Although malnutrition is associated with adverse outcomes in patients with cardiovascular disease, the nutritional status of patients with HF is often overlooked [35, 36]. HF can lead to malnutrition, which, in turn, can lead to increased inflammation, neurohormonal activation, and fluid retention, further affecting HF and forming a vicious cycle [37]. Nutritional assessment is important to clarify the nutritional status of patients and provides a useful predictor of disease risk. nutritional status is thought to influence the prognosis of patients with chronic HF, clarifying the nutritional status of patients for appropriate nutritional interventions can slow the progression of disease and improve the prognosis of patients with HF.

Nutritional screening tools may be affected by recall bias and response rates, and nutritional assessments based on objective biochemical indicators may be more accurate [38–40], such as geriatric nutritional risk index (GNRI) [41], nutritional risk index (NRI) [42] and controlling nutritional status (CONUT) [43]. For hospitalized patients with HF, GNRI and NRI may not be appropriate because increased volume load affects body weight. On the other hand, some HF patients receive statin therapy, which reduces their total cholesterol levels while also possibly affecting the accuracy of the score. Compared to other indicators such as this, PNI, combined with serum albumin level and lymphocyte count, may be a useful screening tool to identify patients at risk of malnutrition. Numerous studies have shown that albumin is a strong predictor of HF prognosis and a valuable tool for assessing nutritional status [44]. However, serum albumin has a long half-life and is also susceptible to nonnutritional factors, such as the patient’s hydration status, infection, abnormal liver function, and nephrotic syndrome. Albumin alone may not provide a complete assessment of a patient’s nutritional status. Lymphocyte count is another determinant of the PNI score. Studies have shown that lymphocyte count is a strong predictor of death in patients with moderate to severe HF [45]. Malnutrition can predispose patients to recurrent infections and cause chronic inflammation. Malnutrition is often associated with an impaired immune response leading to a decrease in lymphocytes [46]. Studies have shown that malnutrition can induce apoptosis in peripheral blood lymphocytes [47].

Undernutrition and overweight have historically been recognized as distinct challenges affecting different populations with different risk factors. Malnutrition encompasses many different manifestations of nutritional deficiencies, including undernutrition and obesity. These two forms of malnutrition are increasingly co-occurring in communities, households and even individuals [48]. Similar to malnutrition, overnutrition may also result from an imbalance between nutritional intake and requirements. Both may disrupt metabolism. Obesity serves as an important aspect of malnutrition. Many studies have shown that obesity, especially excess abdominal fat, is associated with poor health. Delayed nutritional support and increased risk of malnutrition in obese patients may be due to the fact that the nutritional status of such patients is less visible to patients and caregivers. Obesity promotes inflammation, negatively affecting skeletal muscle and metabolic function. The burden of disease caused by obesity and related non-communicable diseases can be a serious public health challenge. With the increase in obesity, the prevalence of MS is also increasing dramatically. The MS is a group of metabolic disorders that includes obesity and is often thought to be the result of excess accumulation of lipids in organs or tissues due to excess nutrients and decreased energy expenditure [49]. This, in turn, disrupts metabolic processes and makes patients vulnerable to metabolic risk factors [20]. Epidemiological evidence defines MS as a highly prevalent worldwide disease [50, 51]. This evidence is further supported by the global increase in overweight and obese populations and their impact on health care systems, economies, and quality of life [52, 53]. In our study, compared with the non-MS group, BMI, SBP, DBP, FPG, TG, and BNP were significantly increased and HCL-C was significantly decreased in malnourished HF patients with MS. At the same time, patients in the MS group had a significantly lower survival rate, and MS was independently associated with the occurrence of adverse events in malnourished patients with HF. Previous studies have shown that the risk of cardiovascular disease increases with increasing components of MS [54]. Similar findings were observed in malnourished patients. In conclusion, the results of our study suggest that malnutrition overlaps with risk factors for metabolic diseases, leading to an increased risk of poor prognosis in patients with HF, and the burdens of overnutrition and undernutrition need to be considered together rather than studied separately.

There are some limitations regarding our study that must be acknowledged. First, it was a single-center cohort study. Although we attempted to adjust for confounders, we cannot completely rule out the risk of bias and residual confounding. Considering the varying degrees of fluid overload in patients with HF, although most patients were treated with diuretic medications, the therapeutic effect was unclear and may have influenced the diagnosis of MS. Also, some patients were followed up by telephone contact with family members, so the results were not completely reliable. Our results do not include other relevant end-point events, and future studies should be designed to further characterize the spectrum of cardiovascular outcomes. The concept of malnutrition in obesity/MS still requires further research and consensus initiatives to better clarify its definition, diagnostic criteria, and treatment options.

## Conclusions

MS is associated with increased cardiovascular death and all-cause death in malnourished HF patients. MS adds incremental value to the prognosis of patients with malnourished HF. For patients who already have a base of malnutrition, MS can impose an even greater burden of malnutrition.

### Electronic supplementary material

Below is the link to the electronic supplementary material.

Supplementary Material 1

## Data Availability

The datasets used and/or analyzed during the current study are available from the corresponding author on reasonable request.
