# Supplementary figures and images for: Prognostic value of metabolic syndrome in patients with heart failure and malnutrition
Source: BMC Cardiovasc Disord. 2024 Mar 2;24:136. doi: 10.1186/s12872-024-03767-5 (PMC10908134; doi:10.1186/s12872-024-03767-5)

Frequency

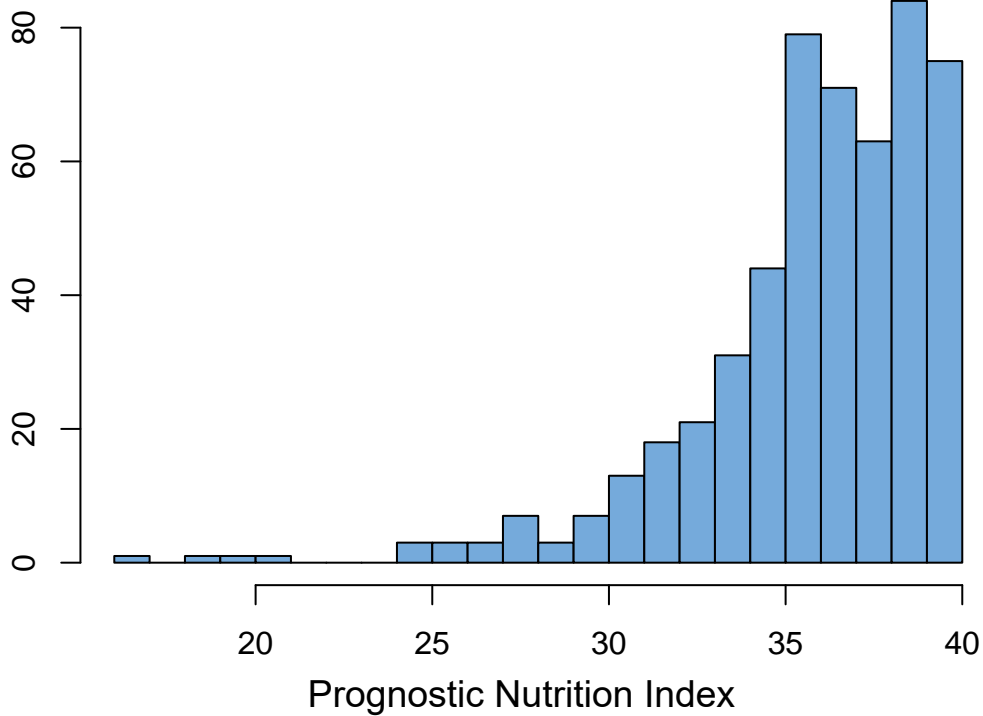

Supplement: Supplementary file 1 — Supplementary Material 1 [file 12872_2024_3767_MOESM1_ESM.pdf]
